# Prognostic value of subclinical thyroid dysfunction in ischemic stroke patients treated with intravenous thrombolysis

**DOI:** 10.18632/aging.102215

**Published:** 2019-09-03

**Authors:** Xiaohao Zhang, Pengyu Gong, Lei Sheng, Yanni Lin, Qiqi Fan, Yun Zhang, Yuanfei Bao, Shizhan Li, Hongcai Du, Zhonglun Chen, Caixia Ding, Huaiming Wang, Pengfei Xu, Min Zhang, Fabien Scalzo, David S. Liebeskind, Yi Xie, Dezhi Liu

**Affiliations:** 1 Department of Neurology, Jiangsu Provincial Second Chinese Medicine Hospital, Second Affiliated Hospital of Nanjing University of Chinese Medicine, Nanjing, Jiangsu, China; 2 Department of Neurology, Jinling Hospital, Medical School of Nanjing University, Nanjing, Jiangsu, China; 3 Department of Neurology, Nanjing First Hospital, Nanjing Medical University, Nanjing, Jiangsu, China; 4 Department of Neurology, The First People’s Hospital of Yulin, Yulin, Guangxi, China; 5 Department of Neurology, Mianyang Central Hospital, Mianyang, Sichuan, China; 6 Department of Neurology, The Third People’s Hospital of Nantong, Nantong, Jiangsu, China; 7 Department of Endocrinology, Nanjing Xianlin Drum Tower Hospital, Nanjing, Jiangsu, China; 8 Department of Neurology, Neurovascular Imaging Research Core, University of California Los Angeles, Los Angeles, CA 90095, USA; 9 Department of Neurology, Shanghai General Hospital, Shanghai Jiao Tong University School of Medicine, Shanghai, China

**Keywords:** subclinical hypothyroidism, subclinical hyperthyroidism, ischemic stroke, thrombolysis, early neurological deterioration

## Abstract

Data regarding the association between subclinical thyroid dysfunction and clinical outcomes in ischemic stroke patients with intravenous thrombolysis (IVT) are limited. We aimed to investigate the predictive value of subclinical thyroid dysfunction in END, functional outcome and mortality at 3 months among IVT patients. We prospectively recruited 563 IVT patients from 5 stroke centers in China. Thyroid function status was classified as subclinical hypothyroidism, subclinical hyperthyroidism (SHyper) and euthyroidism. The primary outcome was END, defined as ≥ 4 point in the NIHSS score within 24 h after IVT. Secondary outcomes included 3-month functional outcome and mortality. Of the 563 participants, END occurred in 14.7%, poor outcome in 50.8%, and mortality in 9.4%. SHyper was an independent predictor of END [odd ratio (OR), 4.35; 95% confidence interval [CI], 1.86–9.68, *P* = 0.003], 3-month poor outcome (OR, 3.24; 95% CI, 1.43–7.33, *P* = 0.005) and mortality [hazard ratio, 2.78; 95% CI, 1.55–5.36, *P* = 0.003]. Subgroup analysis showed that there was no significant relationship between SHyper and clinical outcomes in IVT patients with endovascular therapy. In summary, SHyper is associated with increased risk of END, and poor outcome and mortality at 3 months in IVT patients without endovascular therapy.

## INTRODUCTION

Intravenous thrombolysis (IVT) with recombinant tissue plasminogen activator is an approved medical reperfusion treatment in acute ischemic stroke patients [1]. Recent studies revealed that a substantial fraction of patients cannot virtually recover but experienced early worsening following IVT, which were termed as early neurological deterioration (END) [2]. In fact, END may be accountable for living in dependence and even death, which were not surprisingly found in about half of the stroke survivors, despite IVT [3, 4]. Besides, older age, hyperglycemia, stroke severity, infarct volume, and vascular occlusion have been reported to be associated with a potentially increased risk of poor functional outcome after IVT [3, 5]. However, clinical outcomes are not easily foreseen at the initiation of the therapy as most of the current clinical-radiological risk factors are non-specifc (e.g., advanced age, stroke severity, symptomatic intracerebral hemorrhage, etc.). Therefore, it is essential to detect a potentially useful biomarker, which is involved in the underlying pathophysiological pathway and confers significant predictive value regarding therapy outcomes after IVT.

Subclinical thyroid dysfunction is a common endocrine condition among general population, including a prevalence reaching up to 15% for subclinical hypothyroidism (SHypo), and 12% for subclinical hyperthyroidism (SHyper) [6–9]. The cardio-cerebral vascular system is one of the major targets of thyroid hormones [6, 10, 11]. SHypo has been displayed to propagate vascular risk factors, such as hyperlipidemia [12], metabolic syndrome [13] and vascular stiffness [14]. SHyper has been proved to promote vascular damage by numerous ways, including facilitating hypertension, maintaining hypercoagulable state and causing endothelial dysfunction [15, 16]. Intriguingly, researchers found that ischemic stroke patients with SHypo or SHyper exhibited totally opposite outcomes [17, 18]. SHypo has been reported to be associated with favorable prognosis, while SHyper is related to poor outcomes.

IVT for acute ischemic stroke is often associated with hemorrhagic complications, such as symptomatic or asymptomatic intracerebral hemorrhage, mucosa bleedings and ecchymosis [19]. Thyroid diseases associated with thrombolysis are extremely rare. However, cases of thyroid hemorrhage have been reported [20, 21]. Besides, rt-PA can liberate bradykinin, causing vasodilation and capillary leakage in thyroid [22]. Recently, a case of transient thyroid edema has been confirmed following intravenous thrombolysis for acute ischemic stroke [23]. Thus, we hypothesized that IVT treatment may change the roles of thyroid function in predicting the clinical outcomes of ischemic stroke patients. Several cross-sectional studies have validated the predictive effects of thyroid function on prognosis in ischemic stroke patients [17, 18, 24], while there are yet no data on the prognostic value of subclinical thyroid dysfunction patterns in ischemic stroke patients treated with IVT. Herein, we aimed performed this prospective cohort to identify whether a potential prognostic value was existed in thyroid function in relation to short- and long-term outcomes of IVT patients and whether different dysfunction patterns would possess distinct prognostic values.

## RESULTS

**Figure 1:**
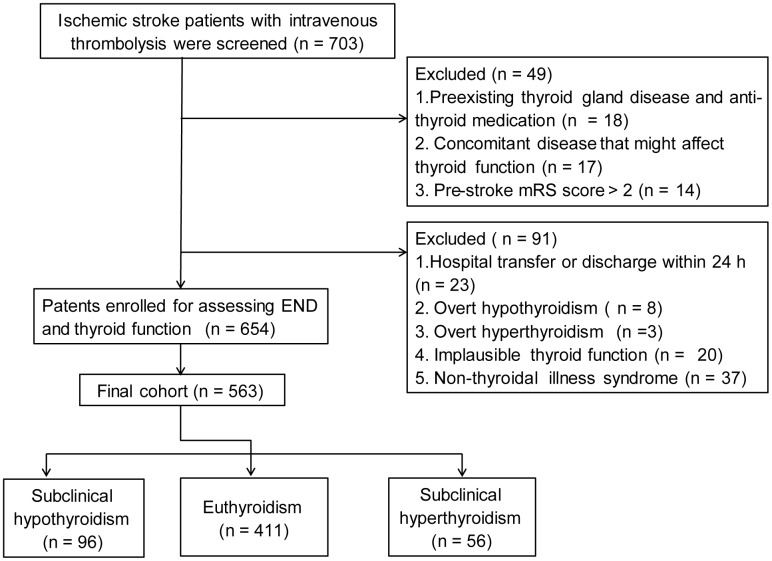
**Flow chart of patient inclusion.**

Finally, 563 ischemic stroke patients (mean age, 67.0 ± 11.8 years; 58.4% male) with thrombolytic therapy were recruited. Flow chart of patient inclusion was present in Figure 1. The baseline characteristics between patients included and patients excluded were shown in Table 1.

**Table 1:** Baseline characteristics of the included and excluded patients.

**Variable**	**Study population (n = 563)**	**Excluded subjects (n = 140)**	***P***
Age, year	67.0 ± 11.8	68.2 ± 10.2	0.270
Male,%	329 (58.4)	92 (65.7)	0.124
Hypertension,%	390 (69.3)	105 (75.0)	0.184
Diabetes mellitus,%	151 (26.8)	38 (27.1)	0.939
Hyperlipidemia,%	72 (12.8)	19 (13.5)	0.507
Coronary heart disease,%	99 (17.6)	28 (19.9)	0.536
Smoking,%	207 (37.0)	59 (42.1)	0.265

After admission, SHypo, SHyper and euthyroidism were found in 96 (17.1%), 56 (9.9%) and 411 (73.0%) patients, respectively. END was observed in 83 (14.7%) patients. Of the 563 patients, 50.8% developed poor outcome, and 9.4% died at 3 months. Compared with euthyroidism, patients with SHypo had a higher prevalence of diabetes and coronary heart disease, higher triglyceride levels, but a lower baseline NIHSS score. Furthermore, hypertension, END, poor outcome and mortality at 3 months were more common in patients with SHyper compared with patients with euthyroidism (Table 2).

**Table 2:** Baseline characteristics of the study population stratified by the status of subclinical thyroid function.

**Variable**	**Euthyroidism group (n = 411)**	**SHyper group (n = 56)**	P^*^	**SHypo group (n = 96)**	***P*^#^**
**Demographics data**					
Age, year	67.3 ± 11.7	65.1 ± 11.2	0.207	66.8 ± 12.9	0.691
Male, %	247 (60.1)	33 (58.9)	0.867	49 (51.0)	0.105
**Vascular risk factors, %**					
Hypertension	283 (68.9)	48 (85.7)	0.009	59 (61.5)	0.164
Diabetes mellitus	105 (25.5)	12 (21.4)	0.505	34 (35.4)	0.049
Hyperlipidemia	50 (12.2)	5 (9.1)	0.477	17 (17.7)	0.155
Coronary heart disease	67 (16.3)	8 (14.5)	0.739	24 (25.0)	0.046
Atrial fibrillation	115 (28.0)	20 (35.7)	0.231	26 (27.1)	0.860
Smoking	157 (39.0)	19 (36.5)	0.806	31 (32.3)	0.273
**Clinical data**					
Previous antiplatelet, %	85 (20.7)	13 (24.5)	0.524	17 (17.7)	0.506
Previous statin, %	34 (8.3)	7 (12.5)	0.294	10 (10.4)	0.502
Systolic blood pressure, mmHg	136.8 ± 20.9	137.5 ± 24.8	0.760	136.2 ± 21.3	0.892
Diastolic blood pressure, mmHg	88.1 ± 14.8	85.4 ± 14.5	0.217	86.2 ± 12.8	0.252
Body mass index, kg/m^2^	24.1 ± 3.2	24.5 ± 3.8	0.366	24.4 ± 3.2	0.451
Onset to blood drawing time, h	11.0 (6.0, 17.0)	11.0 (6.0, 16.0)	0.592	10.0 (6.0, 15.0)	0.277
Baseline NIHSS, score	8.0 (4.0, 13.0)	11.0 (4.0, 19.0)	0.076	5.0 (3.0, 11.0)	0.010
Onset to treatment time, minutes	144.8 ± 61.4	148.7 ± 69.0	0.657	142.6 ± 65.0	0.767
**Imaging data**					
ASPECTS at admission	9.0 (9.0, 10.0)	9.0 (9.0, 10.0)	0.616	9.0 (9.0, 10.0)	0.793
ASPECTS at 24 h	8.0 (6.0, 9.0)	7.0 (5.0, 9.0)	0.061	8.0 (6.0, 9.0)	0.384
Vascular occlusion, %^†^	178 (46.6)	30 (56.6)	0.183	47 (51.1)	0.465
sICH, %	22 (5.4)	6 (10.7)	0.113	2 (2.1)	0.174
**Lesion location, %**			0.467		0.815
Frontal lobe	98 (23.8)	19 (33.9)		19 (19.8)	
Parietal lobe	40 (9.7)	4 (7.1)		7 (7.3)	
Basal ganglia	118 (28.7)	16 (28.6)		31 (32.3)	
Posterior fossa	76 (18.5)	10 (17.9)		19 (19.8)	
Other	79 (19.2)	7 (12.5)		20 (20.8)	
**Stroke subtype, %**			0.116		0.364
Large artery atherosclerosis	161 (39.2)	29 (51.8)		41 (42.7)	
Cardioembolism	107 (26.0)	14 (25.0)		20 (20.8)	
Small vessel occlusion	88 (21.4)	11 (19.6)		26 (27.1)	
Others	55 (13.4)	2 (3.6)		9 (9.4)	
**Clinical outcomes, %**					
Early neurological deterioration	56 (13.6)	19 (33.9)	0.001	8 (8.3)	0.160
Unfavorable outcome at 3 months	205 (49.9)	40 (71.4)	0.003	41 (42.7)	0.206
Mortality at 3 months	33 (8.0)	16 (28.6)	0.001	4 (4.2)	0.190
**Thyroid function tests**					
TSH, mIU/L	1.3 (0.9, 1.7)	0.3 (0.2, 0.4)	0.001	3.4 (2.8, 4.1)	0.001
Free T3, pmol/L	4.8 ± 0.9	4.9 ± 0.9	0.603	4.8 ± 0.9	0.944
Free T4, pmol/L	14.6 ± 2.8	14.6 ± 2.7	0.735	14.1 ± 2.7	0.154
**Laboratory findings**					
TC, mmol/L	4.4 ± 1.1	4.4 ± 1.0	0.911	4.6 ± 1.2	0.278
TG, mmol/L	1.2 (0.9, 1.8)	1.1 (0.8, 1.8)	0.675	1.4 (1.1, 2.3)	0.018
LDL, mmol/L	2.6 (2.1, 3.3)	2.8 (2.1, 3.3)	0.512	2.6 (2.1, 3.2)	0.972
HDL, mmol/L	1.2 ± 0.3	1.2 ± 0.3	0.802	1.2 ± 0.4	0.742
FGB, mmol/L	6.3 ± 2.2	6.4 ± 2.2	0.703	6.5 ± 2.8	0.464
Homocysteine, umol/L	15.7 ± 9.2	16.1 ± 7.4	0.795	15.2 ± 8.0	0.586
Hs-CRP, mg/L	5.7 (2.3, 12.3)	8.4 (2.0, 11.6)	0.388	5.0 (2.0, 12.5)	0.343

Abbreviations: ASPECTS: alberta stroke program early CT score; FGB: fasting blood glucose; HDL: high density lipoprotein; Hs-CRP: high-sensitivity C-reactive protein; LDL: low density lipoprotein; NIHSS: national institutes of health stroke scale; SHyper: subclinical hyperthyroidism; SHypo: subclinical hypothyroidism; sICH: symptomatic intracranial hemorrhage; TC: total cholesterol; TG: triglyceride; TSH: thyroid stimulating hormone.

*Univariate analysis between SHyper group and euthyroidism group;

^#^Univariate analysis between SHypo group and euthyroidism group;

^†^Data available for 525 patients.

Table 3 demonstrated the baseline data of the study population according to clinical outcomes. Patients with END were older, had higher prevalence of endovascular therapy, vascular occlusion, sICH, large artery atherosclerosis and SHyper, and had higher baseline NIHSS score, onset to treatment time, fasting blood glucose and homocysteine levels. The ASPECTS score at 24 h was lower in patients with END than those without. Furthermore, endovascular therapy, vascular occlusion, sICH, and SHyper were more frequent in patients with 3-month poor outcome and mortality.

**Table 3:** Baseline characteristics of the study population stratified by the clinical outcomes in IVT patients.

**Variable**	**END**		***P***	**Poor outcome at 3 months**		***P***	**Mortality at 3 months**		***P***
	**Yes (n = 83)**	**No (n = 480)**		**Yes (n = 286)**	**No (n = 277)**		**Yes (n = 53)**	**No (n = 510)**	
**Demographics data**									
Age, year	69.1 ± 9.6	66.2 ± 11.8	0.007	67.5 ± 11.8	65.7 ± 11.2	0.055	68.7 ± 12.0	66.4 ± 11.5	0.173
Male, %	44 (53.0)	285 (59.4)	0.277	122 (42.7)	112 (40.4)	0.592	27 (50.9)	302 (59.2)	0.245
**Vascular risk factors, %**									
Hypertension	60 (72.3)	330 (68.8)	0.519	94 (32.9)	79 (28.5)	0.264	39 (73.6)	351 (68.8)	0.475
Diabetes mellitus	26 (31.3)	125 (26.0)	0.316	71 (24.8)	80 (28.9)	0.278	13 (24.5)	138 (27.1)	0.692
Hyperlipidemia	10 (12.2)	62 (12.9)	0.857	41 (14.3)	31 (11.2)	0.271	9 (17.0)	63 (12.4)	0.340
Coronary heart disease	18 (21.7)	81 (16.9)	0.292	51 (17.9)	48 (17.3)	0.860	8 (15.1)	91 (17.9)	0.613
Atrial fibrillation	19 (22.9)	142 (29.6)	0.213	90 (31.5)	71 (25.6)	0.125	25 (47.2)	136 (26.7)	0.098
Smoking	32 (39.0)	176 (37.0)	0.723	113 (39.5)	95 (34.9)	0.263	17 (32.1)	191 (37.8)	0.410
**Clinical data**									
Previous antiplatelet, %	21 (25.9)	94 (19.7)	0.197	53 (18.5)	62 (22.8)	0.232	11 (20.8)	104 (20.6)	0.972
Previous statin, %	6 (7.2)	45 (9.4)	0.529	25 (8.7)	26 (9.4)	0.790	5 (9.4)	46 (9.0)	0.904
SBP, mmHg	137.7 ± 18.8	136.7 ± 17.5	0.639	136.9 ± 18.5	136.9 ± 16.9	0.873	138.3 ± 19.4	136.8 ± 17.5	0.524
DBP, mmHg	87.7 ± 11.6	86.2 ± 11.9	0.215	86.8 ± 12.9	85.7 ± 10.9	0.284	87.8 ± 14.6	86.1 ± 11.6	0.316
Body mass index, kg/m^2^	23.8 ± 2.8	24.2 ± 3.3	0.322	24.2 ± 3.2	24.1 ± 3.3	0.675	24.1 ± 4.1	24.2 ± 3.1	0.737
Baseline NIHSS, score	14.0 (6.0, 19.0)	7.0 (4.0, 12.0)	0.001	10.0 (5.0, 16.0)	5.0 (3.0, 10.0)	0.001	16.0 (10.0, 20.0)	7.0 (4.0, 12.0)	0.001
OTT, minutes	158.0 ± 63.1	142.8 ± 62.3	0.047	164.5 ± 59	124.9 ± 60.2	0.002	158.5 ± 57.9	143.4 ± 62.8	0.095
Endovascular therapy, %	21 (25.3)	64 (13.3)	0.005	71 (24.8)	14 (5.1)	0.001	17 (32.1)	68 (13.3)	0.001
**Center, %**			0.389			0.234			0.156
Center 1	42 (50.6)	197 (41.0)		129 (45.1)	110 (39.7)		19 (35.8)	220 (43.1)	
Center 2	9 (10.8)	85 (17.7)		49 (17.1)	45 (16.2)		6 (11.3)	88 (17.3)	
Center 3	12 (14.5)	62 (12.9)		41 (14.3)	33 (11.9)		11 (20.8)	63 (12.4)	
Center 4	11 (13.3)	77 (16.0)		37 (12.9)	51 (18.4)		7 (13.2)	81 (15.9)	
Center 5	9 (10.8)	59 (12.3)		30 (10.5)	38 (13.7)		10 (18.9)	58 (11.4)	
**Imaging data**									
ASPECTS at Admission	9.0 (9.0, 10.0)	9.0 (9.0, 10.0)	0.167	9.0 (9.0, 10.0)	10.0 (9.0, 10.0)	0.205	9.0 (9.0, 10.0)	9.0 (9.0, 10.0)	0.137
ASPECTS at 24 h	6.0 (5.0, 7.0)	8.0 (6.0, 9.0)	0.001	6.0 (5.0, 8.0)	9.0 (7.0, 9.0)	0.001	6.0 (5.0, 7.0)	8.0 (6.0, 9.0)	0.001
Vascular occlusion, %^†^	47 (64.4)	208 (46.2)	0.004	150 (56.8)	105 (40.2)	0.002	35 (66.0)	220 (46.0)	0.001
sICH, n (%)	22 (26.5)	8 (1.7)	0.001	22 (7.7)	8 (2.9)	0.012	16 (30.2)	14 (2.7)	0.001
**Ischemic area, %**			0.689			0.907			0.641
Frontal lobe	20 (24.1)	116 (24.2)		72 (25.2)	64 (23.1)		14 (26.4)	122 (23.9)	
Parietal lobe	8 (9.6)	43 (9.0)		26 (9.1)	25 (9.0)		7 (13.2)	44 (8.6)	
Basal ganglia	23 (27.7)	242 (29.6)		84 (29.4)	81 (29.2)		14 (26.4)	151 (29.6)	
Posterior fossa	15 (18.1)	90 (18.8)		49 (17.1)	56 (20.2)		11 (20.8)	94 (18.4)	
Other	17 (20.5)	89 (18.5)		55 (19.2)	51 (18.4)		7 (13.2)	99 (19.4)	
**Stroke subtype, %**			0.014			0.056			0.072
Large artery atherosclerosis	46 (55.4)	185 (38.5)		122 (42.7)	109 (39.4)		23 (43.4)	208 (40.8)	
Cardioembolism	21 (25.3)	120 (25.0)		78 (27.3)	63 (22.7)		19 (35.8)	122 (23.9)	
Small vessel occlusion	7 (8.4)	118 (24.6)		50 (17.5)	75 (27.1)		5 (9.4)	120 (23.5)	
Others	9 (10.8)	57 (11.9)		36 (12.6)	30 (10.8)		6 (11.3)	60 (11.8)	
**Thyroid function status**			0.007			0.002			0.002
Euthyroidism	56 (67.5)	355 (74.0)		205 (71.7)	206 (74.4)		33 (62.3)	378 (74.1)	
SHyper	19 (22.9)	37 (7.7)		40 (14.0)	16 (5.8)		16 (30.2)	40 (7.8)	
SHypo	8 (9.6)	88 (18.3)		41 (14.3)	55 (19.9)		4 (7.5)	92 (18.0)	
**Laboratory findings**									
TC, mmol/L	4.5 ± 1.0	4.4 ± 1.1	0.636	4.4 ± 1.1	4.5 ± 1.2	0.261	4.2 ± 0.9	4.3 ± 1.1	0.124
TG, mmol/L	1.4 (0.9, 1.9)	1.4 (1.0, 2.0)	0.528	1.3 (0.9, 2.0)	1.5 (1.0, 2.0)	0.312	1.5 (0.8, 2.2)	1.4 (1.0, 2.0)	0.942
LDL, mmol/L	2.7 (2.2, 3.3)	2.6 (2.1, 3.2)	0.174	2.7 (2.1, 3.2)	2.5 (2.1, 3.1)	0.197	2.5 (2.1, 3.1)	2.6 (2.1, 3.2)	0.524
HDL, mmol/L	1.2 ± 0.3	1.1 ± 0.3	0.304	1.2 ± 0.3	1.1 ± 0.3	0.544	1.2 ± 0.2	1.2 ± 0.3	0.847
FGB, mmol/L	7.1 ± 2.6	6.1 ± 2.3	0.003	6.4 ± 2.5	6.3 ± 2.3	0.507	6.8 ± 3.1	6.2 ± 2.3	0.105
Homocysteine, umol/L	18.1 ± 9.5	15.2 ± 8.7	0.011	16.8 ± 8.8	15.8 ± 8.7	0.003	16.0 ± 6.8	15.6 ± 9.1	0.695
Hs-CRP, mg/L	7.9 (2.4, 16.7)	5.1 (2.3, 12.2)	0.102	5.1 (2.1, 11.4)	6.0 (2.8, 12.6)	0.384	10.0 (2.9, 17.8)	5.3 (2.2, 11.0)	0.074

Abbreviations: ASPECTS: alberta stroke program early CT score; FGB: fasting blood glucose; HDL: high density lipoprotein; Hs-CRP: high-sensitivity C-reactive protein; LDL: low density lipoprotein; NIHSS: national institutes of health stroke scale; OTT: onset to treatment time; SHyper: subclinical hyperthyroidism; SHypo: subclinical hypothyroidism; sICH: symptomatic intracranial hemorrhage; TC: total cholesterol; TG: triglyceride.

^†^Data available for 525 patients.

Center 1: Nanjing First Hospital; Center 2: Mianyang Central Hospital; Center3: The First people’s Hospital of Yulin; Center 4: The Third People’s Hospital of Nantong. Center 5: Jiangsu Provincial Second Chinese Medicine Hospital.

Univariate regression analysis demonstrated that SHyper was associated with increased risk of END [odd ratio (OR), 3.26; 95% confidence interval [CI], 1.75–6.06, *P* = 0.001], 3-month poor outcome (OR, 2.51; 95% CI, 1.36–4.63; *P* = 0.003) and mortality [hazard ratio, 3.98; 95% CI, 2.24–7.43, *P* = 0.001]. Furthermore, after adjusting for age, sex, cardiovascular risk factors, center and variables with *P* < 0.05 in univariate analysis, this association did not significantly attenuate. However, no association was found in SHypo with clinical outcomes after IVT (Table 4).

**Table 4:** Unadjusted and adjusted regression analysis for clinical outcomes in IVT patients.

	**OR (95%CI) for END**	***P***	**OR (95%CI) for poor outcome at 3 months**	***P***	**HR (95%CI) for mortality at 3 months**	***P***
Unadjusted model						
SHyper vs Euthyroidism	3.26 (1.75–6.06)	0.001	2.51 (1.36–4.63)	0.003	3.98 (2.24–7.43)	0.001
SHypo vs Euthyroidism	0.58 (0.27–1.25)	0.164	0.75 (0.48–1.17)	0.207	0.51 (0.18–1.44)	0.199
Model 1						
SHyper vs Euthyroidism	3.96 (2.06–7.68)	0.001	3.14 (1.66–5.92)	0.001	3.92 (2.13–7.24)	0.001
SHypo vs Euthyroidism	0.56 (0.25–1.23)	0.146	0.72 (0.46–1.15)	0.167	0.48 (0.17–1.36)	0.166
Model 2						
SHyper vs Euthyroidism	4.35 (1.86–9.68)	0.003	3.24 (1.43–7.33)	0.005	2.78 (1.55–5.36)	0.003
SHypo vs Euthyroidism	0.78 (0.30–2.01)	0.601	0.58 (0.30–1.12)	0.104	0.47 (0.14–1.75)	0.225

Abbreviations: CI: confidence interval; END: Early neurological deterioration; HR: hazard ratio; OR: odd ratio.

Eighty-five (15.1%) patients received endovascular therapy after admission. Results of subgroup analysis according to the IVT patients with or without endovascular therapy were listed in Table 5. For the patients receiving IVT followed by endovascular therapy, SHypo showed a trend for predicting 3-month poor outcome (adjusted OR, 0.27; 95% CI, 0.08–1.06; *P* = 0.057), but not for END and mortality. However, no association was found in SHyper with clinical outcomes among these patients.

**Table 5:** Subgroup analysis according to the IVT patients with and without endovascular therapy.

**Clinical outcomes**	**Patients with endovascular therapy (n = 85)**				**Patients without endovascular therapy (n = 478)**		
	**Euthyroidism (n = 60)**	**SHyper (n = 12)**	**SHypo (n = 13)**		**Euthyroidism (n = 351)**	**SHyper (n = 44)**	**SHypo (n = 83)**
**Early neurological deterioration**							
Unadjusted OR (95%CI)	Reference	1.34 (0.36–5.20)	0.23 (0.03–1.91)		Reference	4.02 (1.99–8.14)*	0.72 (0.31–1.66)
Adjusted OR (95%CI)	Reference	1.17 (0.28–4.95)	0.22 (0.03–1.96)		Reference	4.72 (2.08–9.69)*	1.08 (0.44–2.65)
**Poor outcome** at 3 months							
Unadjusted OR (95%CI)	Reference	1.69 (0.19–9.94)	0.25 (0.06–1.04)		Reference	2.50 (1.30–4.83)*	0.85 (0.30–4.83)
Adjusted OR (95%CI)	Reference	1.40 (0.15–9.33)	0.27 (0.08–1.06)		Reference	2.54 (1.21–5.33)*	0.84 (0.49–1.45)
**Mortality** at 3 months							
Unadjusted HR (95%CI)	Reference	2.75 (0.85–6.54)	0.39 (0.04–2.27)		Reference	4.29 (2.63–9.03)*	0.83 (0.29–2.44)
Adjusted HR (95%CI)	Reference	1.77 (0.58–5.44)	0.37 (0.06–2.31)		Reference	3.99 (1.81–9.30)*	0.89 (0.26–3.12)

Abbreviations: CI: confidence interval; OR: odd ratio; HR: hazard ratio; SHyper: subclinical hyperthyroidism; SHypo, subclinical hypothyroidism.

*P < 0.05

Adjusted model was controlled for age, sICH, ASPECTS score at 24 h and vascular occlusion.

**Figure 2:**
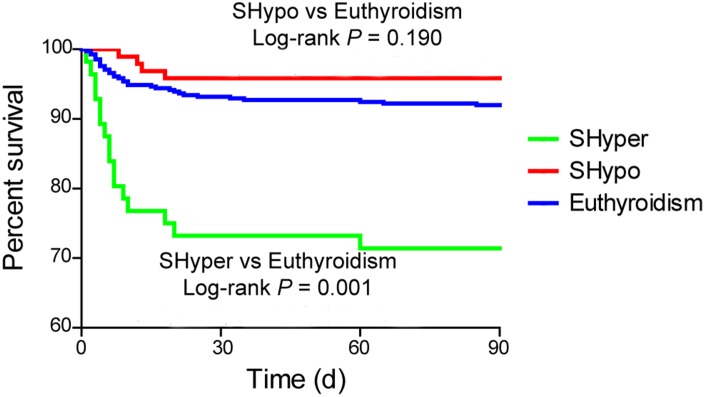
**The Kaplan Meier curve for the cumulative 3-month survival rates according to the thyroid status.** Log-rank test shows significant difference between patients with SHyper and euthyroidism.

Kaplan-Meier curve revealed that mortality at 3 months was significantly higher in patients with SHyper when compared with euthyroidism. No association was detected in mortality between patients with SHypo and euthyroidism (Figure 2, log-rank test, *P* = 0.001).

## DISCUSSION

In this large prospective cohort study of 563 ischemic stroke patients underwent IVT, we demonstrated that SHyper may increase the risk of END, poor outcome and mortality at 3 months in IVT patients without endovascular therapy.

The incidence of 14.7% for END in our study is similar to two recently published series of IVT-treated patients sharing the same END definition [25, 26]. Several risk factors have been postulated to lead to thrombolysis END, such as high blood glucose, hemorrhagic transformation, thrombus extension, and persistent arterial occlusion [25–27]. Data is scarce regarding the effect of neuroendocrine factors on mediating clinical outcomes after IVT. It is now believed that thyroid dysfunction is a potential mediator of the presence and outcome of cerebrovascular diseases. SHypo has been reported to be associated with favorable prognosis, while SHyper related to poor outcomes [17, 18]. Several other studies demonstrate that SHypo and elevated serum TSH level were correlated to increased risk of atherosclerosis [28, 29]. Cases of severe SHypo showed obvious exacerbation of carotid atherosclerosis [28]. In contrast, Cikim et al. found that SHypo was not associated with carotid atherosclerosis, but rather with decreased carotid artery intima-media thickness [30]. Interestingly, our present data did not show a remarkable association of SHypo with large artery atherosclerotic ischemic stroke and vascular occlusion. Some of these discrepancies might be explained at least in part by differences concerning the study population and methodology, especially the assessment of subclinical thyroid dysfunction.

More recently, high TSH levels were demonstrated to be independently correlated with a decreased risk of NIHSS score ≥ 5 at admission (prevalence proportion ratios, 0.62; 95% CI, 0.41–0.94, *P* = 0.024 for 3^rd^tertile vs. 1^st^ tertile) [31]. In addition, patients with high TSH levels may have a better functional outcome at discharge [31]. To our best knowledge, this is the first prospective study that assessed subclinical thyroid dysfunction pattern specifically in relation to clinical outcomes in IVT patients and found that SHyper may increase the risk of END and worse outcome at 3 months. Consistent with our hypothesis, the multivariable logistic regression analysis revealed an independent association between SHyper and END even after adjusting for age, NIHSS score, stroke subtypes, onset to treatment time, sICH, fasting blood glucose levels, homocysteine levels, ASPECTS score at 24 h after thrombolysis and vascular occlusion. There are several mechanisms that might explain for the negative effects of SHyper on worse outcome in patients receiving IVT. Firstly, elevated concentrations of thyroid hormones are associated with an increase in metabolic rate and hyperactive condition in neural tissues, which would be susceptible to the amplitude of neurological damage after ischemia-reperfusion [32]. Secondly, SHyper can cause hypercoagulable state [16, 33], negatively affecting the utility of IVT. Also, pro-inflammatory in SHyper is prone to result in endothelial dysfunction [16], an important pathogenetic role in the progression of stroke and unfavorable outcomes.

In the subgroup analysis of patients treated with IVT followed by endovascular therapy, SHypo showed a protective effect on functional outcome at 3 months. A reduced response under condition of SHypo has been viewed as a protective preconditioning before stroke [7, 17, 24]. Additionally, long-lasting SHypo inducing atherosclerosis in cerebral vessels may also contribute to the development of collateral vessels, which has been widely accepted to be associated with better clinical outcome after endovascular therapy [34]. However, we could not establish an association of SHyper with worse outcome in these patients. These results should be extended and confirmed in future studies.

The strengths of our study include its prospective design and multiple-centers with large sample size. Also, there are limitations to this study that should be considered when interpreting the results. Firstly, some studies have shown the association of neuro-imaging markers such as thrombus extension, persistent arterial occlusion and vascular recanalization with END. However, these markers were not measured in this study, and we did not exclude effects of these markers on END. Also, we cannot totally exclude the potential effect of other undetected factors such as drug, stress induced by stroke, disease status and genetic predisposition on the level of thyroid function. Secondly, FT3, FT4, and TSH concentrations were measured only once at admission. A serial test would also be useful in verifying the role of thyroid function as a predisposing factor on acute stroke outcomes. Finally, several patients transfer to other hospitals or discharge early, presumably because of severe neurological deficit. Exclusion of such patients might have underestimated the prevalence of END and poor outcome.

In conclusion, this study illustrates that SHyper might be associated with increased risk of END, 3-month poor outcome and mortality in IVT patients without endovascular therapy. The potential benefits of screening and intervention of thyroid function on early clinical recovery require further exploration in ischemic patients underwent IVT.

## MATERIALS AND METHODS

### Study population

From January 2017 to March 2018, we consecutively recruited acute ischemic stroke patients underwent IVT within 4.5 h of symptom onset at 5 stroke centers in China (Nanjing First Hospital, Mianyang Central Hospital, The First people’s Hospital of Yulin, The Third People’s Hospital of Nantong, and Jiangsu Provincial Second Chinese Medicine Hospital). Patients treated with a bridging therapy consisting of IVT followed by endovascular therapy were also included. Exclusion criteria were as follows: (1) hospital transfer or discharged within 24 h; (2) pre-stroke modified Rankin Scale (mRS) score > 2; (3) pre-existing thyroid glands diseases, anti-thyroid medications, or diseases that might affect thyroid function [35, 36]; (3) defined as any of the following thyroid function alterations after admission: implausible thyroid function, biochemically defined overt thyroid disease, and non-thyroidal illness syndrome [37]. All participants signed a written consent form, and the study protocol was approved by the Institutional Review Board at each hospital.

### Clinical data collection and assessment criteria

Data collection was performed by neurologists with experience in stroke care using a standardized case report form. We recorded baseline characteristics, including demographics, cardiovascular risk factors (including hypertension, diabetes, hyperlipidemia, smoking, drinking, and atrial fibrillation) and previous medication. Moreover, body mass index, blood pressure, onset to-treatment time, imaging data, and stroke subtype were also recorded. In this study, symptomatic intracerebral hemorrhage (sICH) was defined according to the European Cooperative Acute Stroke Study II (ECASS-II) trial [38]. All computed tomography images at admission and 24 h after IVT were calculated for the Alberta Stroke Program Early CT Scores (ASPECTS) [39]. Computed tomography angiography, magnetic resonance angiography or digital subtraction angiography was performed to identify the affected vessel. Stroke subtype was classified according to TOAST (Trial of Org 10172 in Acute Stroke Treatment) criteria [40].

### Thyroid function tests

Blood samples were obtained from all patients within 24h after onset, and collected in chemistry test tubes. After centrifugation, serum samples were separated, and kept frozen at −80 °C for later analysis. Free triiodothyronine (FT3), free thyroxine (FT4), and thyroid stimulating hormone (TSH) levels were determined for each patient by electrochemiluminescence immunoassay (Abbott Architect i2000, Abbott Diagnostics, Abbott Park, IL, USA). In accordance with previous studies [9, 18], levels of thyroid function were defined as SHyper [0.1 < TSH < 0.45 mIU/L], and SHypo (TSH: 2.50–19.99 mIU/L) with thyroid function test results. Patients with serum TSH levels between 0.45–2.49 mIU/L were considered as control group. Reference ranges for FT4 were 11.58–23.16pmol/L and for FT3 were 3.54–6.46pmol/L.

### Clinical outcomes measurement

The primary outcome was END, which was defined as an increment of at least 4 point in total NIHSS score between admission and within 24 h after IVT (2). During a follow-up at 3 months after the index stroke, all patients were assessed for clinical outcome using the modified Rankin Scale (mRS) by neurologists who were blinded to clinical data. The following secondary outcomes were recorded: (1) functional outcome: categorized as good (mRS score of 0–2) and poor (mRS score of 3–6); (2) mortality.

### Statistical analysis

All statistical analysis was done with SPSS software, version 23.0 (SPSS Inc., Chicago, IL). Continuous variables were summarized as mean ± standard deviations (SD) or medians with interquartile ranges (IQR) and compared by Student's t test or Mann-Whitney U test as appropriate. Categorical variables were expressed as percentages and analyzed with the chi-square test or Fisher’s exact test, as appropriate. Binary logistic regression analysis was designed to describe adjusted estimates of the association of subclinical thyroid dysfunction with END and poor outcome at 3 months. Cox’s proportional hazard regression analysis was performed to estimate the hazard ratio for mortality. All regression analyses were first adjusted for age, sex, cardiovascular risk factors and center (Model 1), and additionally adjusted for variables with *P* < 0.05 in univariate analysis (Model 2). We also used Kaplan-Meier curve to calculate 3-month survival probabilities. In all analyses, *P* < 0.05 was considered as statistically significant.
